# Neuroprotective Effect of HIIT against GFAP Hypertrophy through Mitochondrial Dynamics in APP/PS1 Mice

**DOI:** 10.1155/2022/1764589

**Published:** 2022-02-02

**Authors:** Qianqian Liu, Xiaonan Fu, Rui Han, Xuefeng Liu, Xiantao Zhao, Jianshe Wei

**Affiliations:** ^1^School of Physical Education and Sport, Henan University, Kaifeng, China; ^2^School of Life Sciences, Henan University, Kaifeng, China

## Abstract

Alzheimer's disease (AD) is characterized by the accumulation of *β*-amyloid (A*β*) plaques and tau neurofibrillary tangles in the brain. Although the exact details of the neuronal protective effect of high-intensity interval training (HIIT) on AD remain unclear, the preclinical phase of AD appears to be the important time point for such intervention. The described experiment investigates the neuroprotective effect of HIIT on AD in APP/PS1 mice. In total, 14 C57BL6 healthy control (C) mice and 14 APP/PS1 AD mice were each randomly assigned into two groups, one that did not participate in HIIT (C and AD groups, respectively) and the other subject to HIIT intervention (control HIIT (CE) and AD HIIT (ADE) groups, respectively). Visualization of hippocampal neuronal cells via HE and Congo red staining showed significant improvement in cell status and a significant reduction in amyloidosis in ADE compared with AD. The results of behavioral analysis show that the HIIT intervention significantly improved cognitive decline and reduced spatial exploration in both the C and AD groups. Immunofluorescence showed that the overall brain and the hippocampus of aged rats in the C and AD groups had different degrees of neuroglial responses and astrocyte GFAP proliferation and hypertrophy, with obvious improvement in the CE and ADE groups after 10 weeks of HIIT intervention. These results show that HIIT significantly improves the status of mitochondrial kinetic proteins and related proteins, with the mechanism differing between the normal aging C and the AD groups. 10 weeks of HIIT improved the imbalance in mitochondrial dynamics present in normal control mice and in AD mice. We conclude that preclinical training intervention has a significant positive effect on the exploratory behavior and cognitive functioning of mice.

## 1. Introduction

Alzheimer's disease (AD) is the main cause of dementia, which seriously impacts human health, and the incidence is rising along with the increase in life expectancy. Modern medicine considers AD to be a severe neurodegenerative disorder associated with specific histopathological markers, such as extracellular deposition of A*β*, intracellular aggregates of tau proteins/neurogenic fibrillary tangles [1, 2], and neurodegeneration [3, 4]. However, the pathological manifestations of AD precede the clinical symptoms, and, therefore, if regular exercise and body activity are provided during the preclinical stage, the risk of aggravating the progression of AD may be reduced. Thus, the preclinical phase is considered a critical period for intervention.

Studies have demonstrated that regular physical activity can delay or assist in the avoidance of neurological and psychiatric disorders and is also effective in treating neurological disorders [5–7]. HIIT is a method of training that involves short, repeated bursts of intense exercise lasting from a few seconds to a few minutes, interspersed with low-intensity exercise or rest [8, 9]. HIIT can be an effective alternative to traditional endurance training and induce similar or even better physiological adaptations in both healthy individuals and diseased populations. Studies have shown that high-intensity interval exercise is more effective than traditional aerobic exercise in promoting brain health and enhancing cognitive functioning in the brain [10, 11]. Studies have shown [12] that impaired mitochondrial dynamics is an important early event in AD pathogenesis, and a growing number of studies have demonstrated [13, 14] that HIIT induces altered mitochondrial dynamics and mitochondrial autophagy and plays an important role in maintaining mitochondrial homeostasis.

Studies have concluded that physical activity improves brain metabolism and cognitive function, but the brain region most significantly affected by exercise is the hippocampus [6, 15–17]. The hippocampus is a key structure in the brain that is responsible for learning and memory [18], and appropriate exercise is responsible for an increased volume of blood flow to the hippocampus [17, 19, 20]. Although studies have shown that HIIT is an effective form of physical training, the neuroprotective effects on the hippocampus are not well understood. Mitochondria play a key role in the pathogenesis of AD [21], and in AD rats, hippocampal mitochondria are highly susceptible to damage and abnormal division/fusion. Thus, the exact mechanism by which HIIT provides neuroprotective effect in the context of AD is unclear.

In this paper, we observed the effects of 10-week HIIT on hippocampal neuroprotection and mitochondrial kinetic proteins in C57BL6 mice and APP/PS1 AD mice and discuss the role of HIIT in improving preclinical AD.

## 2. Materials and Methods

### 2.1. Animals

The animal experimental content and exercise protocol were approved by the Ethics Committee of Henan University (approval number: HUSOM2021-138). In total, 14 male C57BL6 mice (7 months old 27 ± 1.0 g) and 14 male APP/PS1 mice (7 months old 27 ± 1.0 g) were provided by Speford (Beijing) Biotechnology Co., Ltd (license number: SCXK(Beijing)2019-0010). SPF-grade national standard rodent feeding pellets and bedding were purchased from the Henan Provincial Laboratory Animal Center (license number: SCXK(YU)2015-0005). During the experiment, the mice were free to drink water, the bedding was changed 2-3 times a week, and the ambient temperature was 20°C-22°C, with natural light and no humidity control.

### 2.2. Animal Grouping and Exercise Program

The mice were randomly and evenly divided into four groups (*n* = 7 in each group) after one week of adaptive feeding: (1) control (C) group of healthy, normal aging C57BL6 mice, (2) control high-intensity interval training (CE) group of C57BL6 mice with HIIT intervention, (3) Alzheimer's disease (AD) group of APP/PS1 mice, and (4) AD with high-intensity interval training (ADE) group of APP/PS1 mice with HIIT intervention. The CE and ADE high-intensity interval training groups underwent 10 days of acclimatization exercise training before the formal training, for which an experimental animal treadmill was used (FT-200, Chengdu Taimeng Technology). The formal training program was as follows: the mice were first subjected to an incremental load test, warming up at 12 m/min for 15 minutes; then, high-intensity interval training was performed, with each HIIT session consisting of 3 min at a 23 m/min incline of 15° and a 1 min rest, for a total of 10 sets. Training was conducted 5 days per week with 2 days of rest for 10 weeks. At the end of 10 weeks of exercise, two tests were performed for behavioral assessment: the open-field and elevated plus maze tests.

### 2.3. Sampling

Experimental animals were executed by overanesthesia after completion of the corresponding interventions in each group. In order to avoid the acute effects of exercise, the sampling was performed after 24 h of rest and 12 h of fasting following the last exercise. Prefixation sampling of mice for morphological testing was undertaken, whereby the whole brain tissue was fixed in neutral formaldehyde for 48 h. For others, the brain tissue was removed from the ice, and the bilateral hippocampus was peeled off within 1 min, placed in a lyophilization tube, snap frozen in liquid nitrogen, and then transferred to a freezer for storage at -80°C.

### 2.4. Histomorphological Observation

The hippocampal region was routinely sectioned and morphologically observed with HE staining and Congo red staining and GFAP immunofluorescence staining.

(1) *HE Staining*. The whole dyeing process includes the following: (1) xylene (I) 15 min, (2) xylene (II) 15 min, (3) xylene : absolute ethanol = 1 : 1 2 min, (4) 100% ethanol (I) 5 min, (5) 100% ethanol (II) 5 min, (6) 80% ethanol 5 min, (7) distilled water 5 min, (8) hematoxylin semen staining 5 min, (9) water washing 10 min or running water washing 5 min, (10) 1% hydrochloric acid ethanol 30 s, (11) water washing 30 s, (12) distilled water over washing 5 s, (13) dyeing with 0.5% eosin solution for 1-3 min, (14) slightly washing with distilled water for 30 s, (15) slightly washing with 80% ethanol for 30 s, (16) 95% ethanol (I) for 1 min, (17) 95% ethanol (II) for 1 min, (18) absolute ethanol (I) for 3 min, (19) absolute ethanol (II) for 3 min, (20) xylene (I) for 3 min, (21) xylene (II) for 3 min, and (22) neutral gum sealing.

(2) *Congo Red Staining*. Paraffin section dewaxing to water: sequentially put the section into xylene I for 20 min, xylene II for 20 min, anhydrous ethanol I for 5 min, anhydrous ethanol II for 5 min, and 75% alcohol for 5 min, tap water washing. Congo red staining: sections were stained with Congo red A solution overnight, washed with tap water for 2 min. Background differentiation: sections were differentiated with Congo red B solution for 1 s until the positive plaque was obvious and the background was basically colorless, washed with tap water. Nuclei staining: sections were stained with Congo red C solution for 1 min, washed with tap water, differentiated with differentiation solution, washed with tap water, returned to blue with blue return solution, and rinsed with running water. Dehydration and sealing: the sections were put into anhydrous ethanol I for 5 min, anhydrous ethanol II for 5 min, anhydrous ethanol III for 5 min, xylem I for 5 min, and xylem II for 5 min transparent, and neutral gum was used to seal the sections. Microscopic examination, image acquisition, and analysis were used.

(3) *GFAP Immunofluorescence Staining*. Dewaxing and rehydration were performed conventionally. Antigen repair is as follows: high temperature water bath. Then, the specimens were blocked by 3% BSA blocking solution for 15 minutes. Add primary antibody: the specimens were incubated with the primary antibodies against the following proteins: GFAP (GB11096): all antibodies were diluted by a factor of 1 : 80 and incubated overnight at 4°C in the wet box. On the next day, the specimens were put into water bath for 30 minutes, followed by washing with 0.01M PBS 5 minutes∗3 times. The specimens were further incubated with fluoresce inisothiocyanate-labeled secondary antibodies (dilution 1 : 50) for 30 minutes. DAPI counterstained nuclei, quenched tissue autofluorescence, seal, and microscopic examination and photography were used.

### 2.5. Behavioral Testing

Behavioral testing was performed using the open-field test and the elevated plus maze. For the open-field test, the mice were placed in the center of an open field in a quiet environment, with the activity of the mice in the open field being filmed with a camera for 5.5 minutes. Following the completion of each test, the inner wall and bottom surface of the open field were thoroughly cleaned to prevent interference with the next test. The mice were sequentially replaced until all mice had been tested. For the elevated plus maze, the mice were placed in the central area of an elevated plus maze, with a video recording of the free movement of the mice with a camera for 6 minutes. Following the completion of each test, all areas were cleaned, and the next mouse was tested until mice had been tested. Behavioral data analysis was conducted using the animal behavior software Smart 3.0.

### 2.6. Western Blotting

Each mouse hippocampus was taken for the extraction and quantification of cytoplasm and mitochondrial proteins, and the protein samples of each group were subjected to resolution in a 4% gel with a constant voltage of 80 V for 30 min, followed by a constant voltage of 150 V for 70 min and then a constant current of 300 mA for 120 min in a 12–15% separation gel. An amount of 5% skimmed milk powder was used for blocking at 4°C for 6 hours and incubation with the primary antibody overnight and then transferred to a PVDF membrane. The membrane was incubated with secondary antibody at 1 : 1000 dilution for 90 min at room temperature. The membranes were rinsed 3 times with TBST for 10 min each and then finally rinsed 2 times with TBS for 10 min each. The target proteins were detected using ECL luminescence. The corresponding grayscale values were read, with *β*-actin as an internal reference for the cytoplasm proteins and COX IV for the mitochondrial proteins, and the relative grayscale values of the target proteins were calculated. Details of the antibodies used are provided below (Table 1).

### 2.7. Statistical Analysis

The SPSS 17.0 software was used to analyze the data, Image-Pro plus 6.0 software was for image analysis, and GraphPad Prism software was for graphing. Data were expressed as mean (±SD) and analyzed using two-way analysis of variance (gene × HIIT). If the interaction between the factors was significant (*p* < 0.05), then the simple effect test was performed. A *p* value of 0.05 or less was considered statistically significant (*p* < 0.05 and *p* < 0.01 indicated significant difference). The data variables were subject to correlation analysis using Pearson correlation analysis.

## 3. Results

### 3.1. Histopathological Changes in the Hippocampus

**Figure 1:**
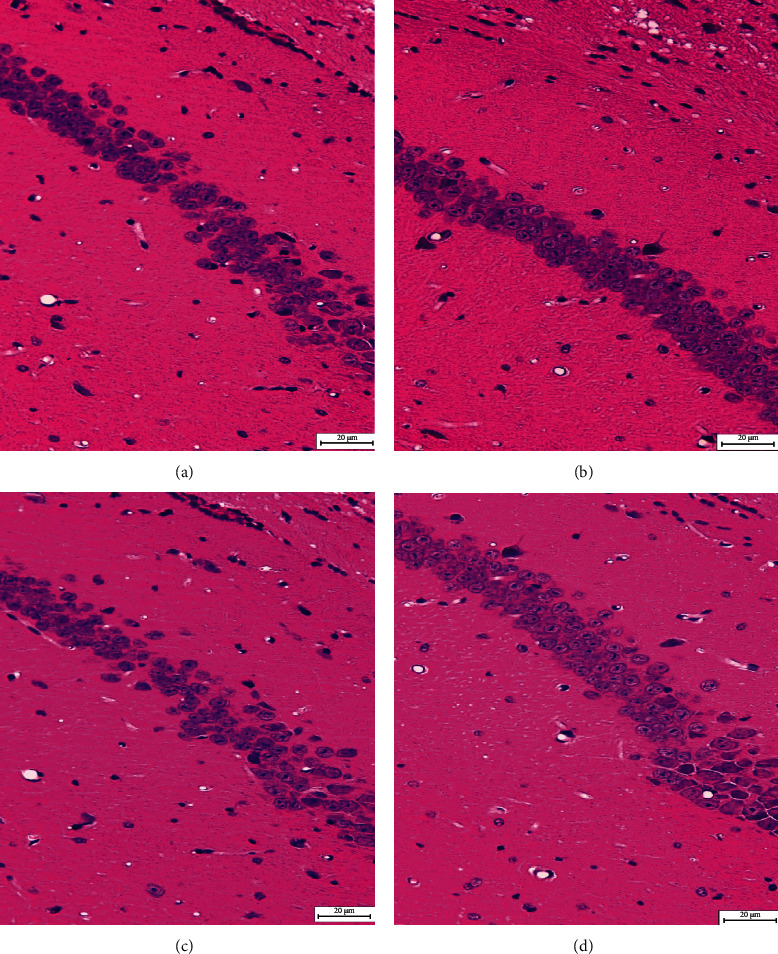
Histopathological changes in the hippocampus tissues (hematoxylin and eosin, scale length = 20 *μ*m). (a–d) Images of the hippocampal CA1 region of C group, CE group, AD group, and ADE group, respectively.

As shown in Figure 1, the glial cells and the hippocampus neurons in the C group are arranged in an orderly manner, the neuronal cells are not quite full, the nucleoli are clear, and the nerve cells are clearly and uniformly stained. The hippocampus nerve cells in the CE group were round and full, the cells were neatly arranged, and the nucleus was round and clear. In the AD group, an obvious decrease in the number of hippocampus nerve cells is observed, and nerve cells are disorderly arranged, with scattering of psychotic cells that have no clear nucleolus boundary. In comparison with the AD group, there was a clear improvement in the ADE group when considering the number of hippocampus nerve cells, nuclear condensation, glial and neuronal cell necrosis, and status of cell structure disruption.

### 3.2. Congo Red Staining of the Hippocampus

The main effect of HIIT on the mean optical density of A*β* was of statistical significance (*p* < 0.01). The main effect of gene on the variable of histology was of statistical significance (*p* < 0.01). Moreover, the mean optical density of A*β* showed interaction effect between HIIT and gene (*p* < 0.01).

**Figure 2:**
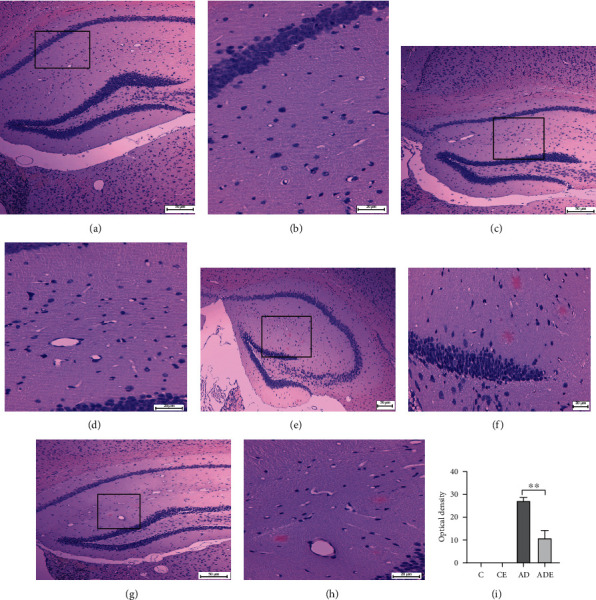
Congo red staining of the hippocampus. (a, b) The C group hippocampus showing normal hippocampus neuron cells (Congo red, 200x and 400x); (c, d) the CE group hippocampus showing normal hippocampal histology and normal hippocampus nerve cells in (Congo red, 200x and 400x); (e, f) the AD group showing the overall structure of the hippocampus, with multiple *β*-amyloid deposits (Congo red, 200x) and multifocal, large-area *β*-amyloid deposition (Congo red, 400x); (g, h) the AD HIIT group showing the overall structure of the hippocampus, beta amyloid deposition reduction (Congo red, 200x), and *β*-amyloid deposition decrease (Congo red, 400x) compared with the AD control (AD) group; (i) Congo red staining of the hippocampus. Data are expressed as mean ± SD, ^∗∗^*p* < 0.01, compared with the AD group.

Congo red staining showed that the nuclei of the hippocampus neurons were round or nearly round. It was also observed that there were no (or rarely) pink plaques in the hippocampus of the mice in either the C group or the CE group. The hippocampuses of nontransgenic and APP/PS1 mice were observed by staining with Congo red dye after 10 weeks of HIIT to detect the deposition of amyloid beta plaques. As expected, the AD group mice accumulated considerable amyloid beta in the hippocampuses, as shown in Figures 2(e) and 2(f). Irregularly shaped pink plaques can be seen in the cytoplasm or extracellular and in large numbers and volumes, as shown in Figure 2(f). The mice that underwent HIIT exhibited a reduction in the amounts of *β*-amyloid in the hippocampus, and the number of pink plaques decreased with small volume changes, as shown in Figures 2(g) and 2(h).

### 3.3. Open-Field Test

The main effect of HIIT on the total distance traveled, maximum speed, and mean speed was of statistical significance and with no significant difference for parallelism index and turning tendency (*p* = 0.021, *p* = 0.009, *p* = 0.021, *p* = 0.005, and *p* = 0.359, respectively). However, the main effect of gene on all variables of behavioral in the open field was not statistically significant (*p* = 0.887, *p* = 0.973, *p* = 0.944, *p* = 0.293, and *p* = 0.893, respectively). Moreover, the total distance traveled, maximum speed, mean speed, parallel index, and turning tendency also showed no interaction effect between HIIT and gene (*p* > 0.05).

**Figure 3:**
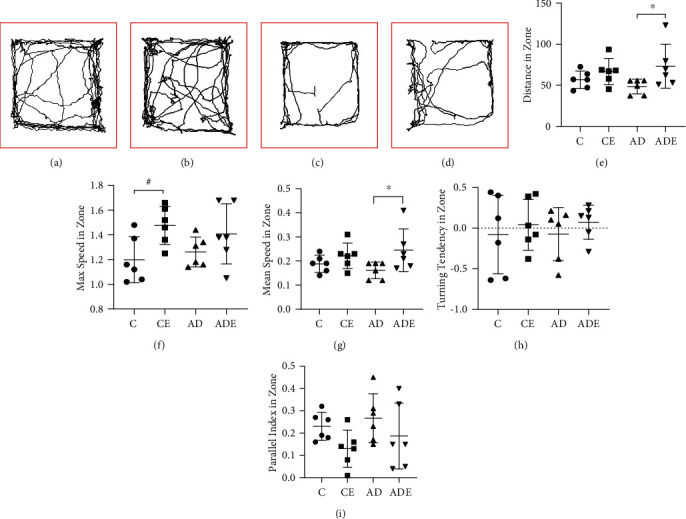
Open-field test. HIIT reversed age or transgenic induced abnormality or impairment of voluntary movement. (a–d) Sample traces of open-field paths from mice in each group; (e) total distance traveled in open field; (f) maximum speed in open field; (g) mean speed in open field; (h) parallel index in open field; (i) turning tendency in open field. The experiment was conducted in each 5 min time period. Values are represented as the mean ± SD (*n* = 6). ^∗^*p* < 0.05 compared with the AD group; ^#^*p* < 0.05 compared with the C group.

Figure 3 shows the changes in locomotor activity and exploratory behavior for each group. Figures 3(a)–3(d) show that, after 10 weeks of HIIT, the expression of exploratory behavior showed an upward trend in the CE and ADE groups, with an increase in the number of crossings in the central area of the open field during exploration. As shown in (e), the analyses showed no change in the total distance of spatial activity between the C and CE groups, but the ADE group showed a significant increase compared with the AD group (*p* < 0.05). The CE group, shown in (f), exhibited a significant increase (*p* < 0.05) in maximum speed compared with the C group, and an increase was observed in the ADE group compared with the AD group, but this difference was not significant. No change in the mean speed was observed between the C and CE groups, but there is a significant increase in the ADE group compared with the AD group (*p* < 0.05). The parallel index in zone decreased in both CE and ADE groups, when compared with the respective control groups, but the difference was not significant. The turning tendency in zone was improved in both the CE and ADE groups compared with the respective control groups, but the difference was not significant.

### 3.4. Elevated Plus Maze

The main effect of HIIT on the open arms entries, time in open arms(%), entries in open arms and close arms, and distance in open arms (%) was of no statistical significance (*p* > 0.05). The main effect of gene on all variables of elevated cross maze test was of statistical significance (*p* < 0.01). Moreover, all variables of elevated cross maze test showed no interaction effect between HIIT and gene (*p* > 0.05).

**Figure 4:**
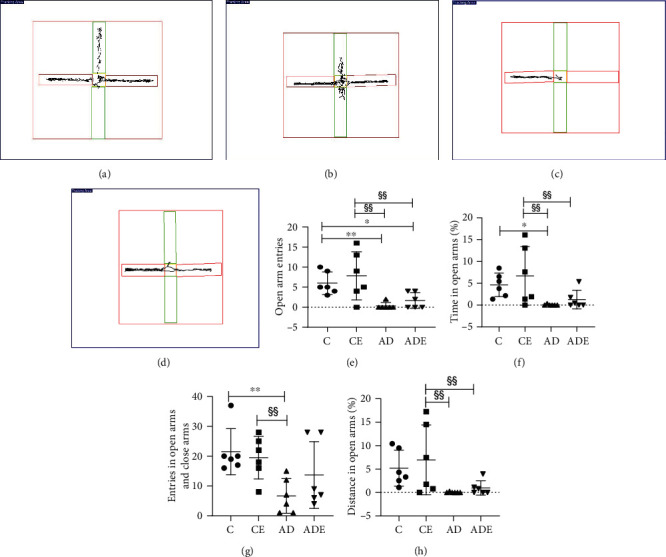
Elevated plus maze. Depression, anxiety-related behavior, and activity measures for the elevated plus maze test. (a–d) Sample traces of elevated plus maze paths from mice in each group; (a–d) are C group, CE group, AD group, and ADE group, respectively. (e–h) Comparison of open arm entries, time in open arms (%), entries in open arms and close arms, and distance in open arms (%) values in the elevated cross maze test of rats in different groups. ^∗∗^*p* < 0.01 and ^∗^*p* < 0.05 compared with the C group; ^§§^*p* < 0.01 compared with the CE group.

Figure 4 shows the changes in the four groups of mice in the elevated cross maze. The expression of exploratory behavior in the CE and ADE groups was significantly increased after 10 weeks of HIIT. The number of exploratory behaviors and the dwell time in the open arms was increased in the CE and ADE groups due to the 10 weeks of high-intensity interval training. However, the intervention effect showed a large intragroup difference, and there is no significant difference between the control groups and corresponding HIIT groups. After 10 weeks of HIIT, the number of open arm explorations increased slightly in the CE group compared with the C group and the ADE group compared with the AD group, with no difference (Figure 4(e)), but the AD group decreased very significantly (*p* < 0.01), and the ADE group declined significantly (*p* < 0.05) compared with the C group; meanwhile, compared with CE group, AD group and ADE group decreased significantly (*p* < 0.01). There was a rise in the time spent in the open arms in the CE and ADE groups compared with the control groups, which shows a significant decrease in the AD group compared with the C group and a significant decrease in both the AD and ADE groups compared with the CE group compared with the control groups, but the AD group shows poor performance and insignificant activity compared with the C and CE groups (*p* < 0.01) (Figure 4(f)). The distance of open arm activity increased in the CE and ADE groups (Figure 4(h)) compared with the respective control groups, but the difference was not significant, and compared with the CE group, the AD and ADE groups show less distance in open arms, with significant difference (*p* < 0.01). The number of entries into the open and closed arms was slightly lower in the CE group than in the C group, but the difference was not significant; however, the number of entries in the ADE group was higher than in the AD group, but, again, the difference was also not significant.

### 3.5. HIIT Alters Hippocampal GFAP Expression in each Group

The main effect of HIIT on the mean optical density of GFAP was of statistical significance (*p* < 0.01). The main effect of gene on the variable of hippocampus GFAP expression was of statistical significance (*p* < 0.01). Moreover, the mean optical density of GFAP showed interaction effect between HIIT and gene (*p* < 0.01).

**Figure 5:**
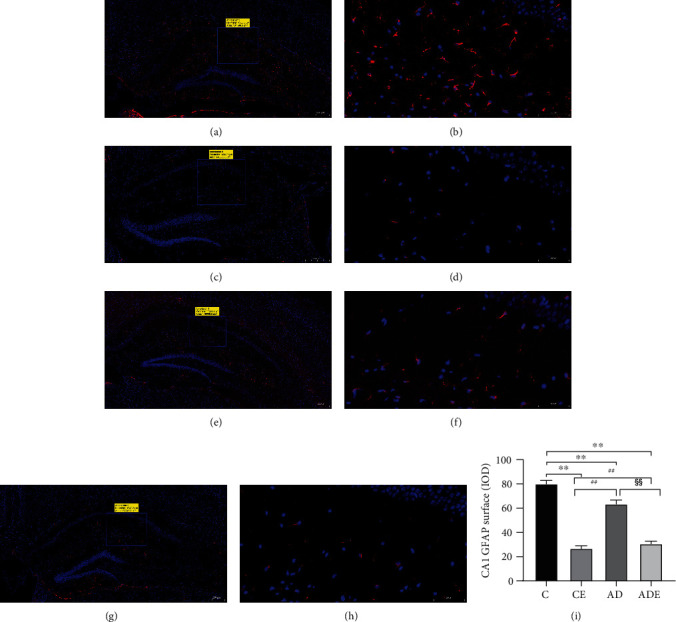
Hippocampus GFAP immunofluorescence. (a, c, e, g) and (b, d, f, h) are C, CE, AD, and ADE groups, respectively. (i) Comparison of CA1 GFAP surface IOD values in different groups. Values are represented as the means ± SD (*n* = 3). ^∗∗^*p* < 0.01 compared with the C group; ^##^*p* < 0.01 compared with the CE group; ^§§^*p* < 0.01 compared with the AD group.

Figure 5 shows immunofluorescent brain slices displaying varying degrees of glial response in the hippocampus and cerebella cortex in each group; GFAP appears proliferated and hypertrophied in the C and AD groups. After 10 weeks of HIIT intervention, more significant improvements were seen in the CE and ADE groups (Figures 5(a), 5(c), 5(e), and 5(g)). As shown in Figure 5(i), there was a significant decrease in the CA1 regions of the hippocampus in CE, AD, and ADE groups compared with the C group, with a highly significant difference (*p* < 0.01). The expression of GFAP was significantly increased in the AD group compared with the CE group, with a significant difference (*p* < 0.01), and the fluorescence expression was also increased in the ADE group compared with the CE group, with the same significant difference (*p* < 0.01). The CA1 regions of the hippocampus showed a significant decrease in the ADE group compared with the AD group, and the difference was highly significant (*p* < 0.01).

### 3.6. HIIT Alters the Expression of Hippocampal Mitochondrial Kinetic Proteins and Related Proteins

The main effect of HIIT on the expression of MFN1, DRP1, p-TAU, FIS1, and MFN2 proteins in the hippocampus mitochondria was of statistical significance (*p* < 0.05); however, the main effect of HIIT on the expression of OPA1 was not statistically significant (*p* > 0.05). The main effect of gene on the variable of hippocampus protein expression was of statistical significance (*p* < 0.05). There was also an interaction effect between HIIT and gene (*p* < 0.05).

**Figure 6:**
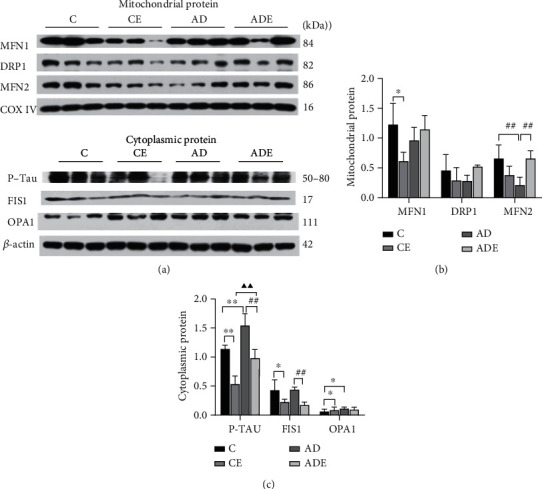
Expression of (a) mitochondrial kinetic proteins and (b, c) related proteins in the hippocampus. Comparison of MFN1, DRP1, MFN2, p-TAU^ser214^, FIS1, and OPA1 values in different groups. Values are represented as the means ± SD. ^∗^*p* < 0.05 and ^∗∗^*p* < 0.01 compared with the C group; ^##^*p* < 0.01 compared with the CE group; ^▲▲^*p* < 0.01 compared with the CE group.

Figure 6(a) shows the expression of MFN1, DRP1, and MFN2 proteins in the hippocampus mitochondria of the mice in each group. MFN1, DRP1, and MFN2 protein expression was reduced in the CE group compared with the C group, whereby the difference in the expression of MFN1, but not DRP1 and MFN2, between the groups was significant (*p* < 0.05). MFN1, DRP1, and MFN2 expression was increased in the ADE group compared with the AD group, and this increase was only significant in the case of MFN2 protein expression (*p* < 0.01). The C group compared with the AD group, the expression of MFN2 in AD group decreased significantly (*p* < 0.01).

Figure 6(b) displays the expression of hippocampus cytoplasm proteins FIS1, OPA1, and phosphorylated tau in the mice in each group. The expression of p-TAU^ser214^ was decreased in the CE group compared with the C group (*p* < 0.01), and this expression trend was the same when comparing the ADE group with the AD group (*p* < 0.01). p-TAU expression was increased in the AD group compared with the C group, which shows significant difference (*p* < 0.01); meanwhile, the expression of p-TAU^ser214^ was increased in the ADE group compared with the CE group (*p* < 0.01). FIS1 expression was significantly decreased in the CE group compared with the C group (*p* < 0.05) and similarly significantly decreased in the ADE group compared with the AD group (*p* < 0.01). OPA1 expression was significantly increased in CE and AD groups compared with the C group (*p* < 0.05) but slightly decreased in the ADE group compared with the AD group, but not significantly.

### 3.7. Correlation Analysis between Mitochondrial Kinetic Proteins

**Figure 7:**
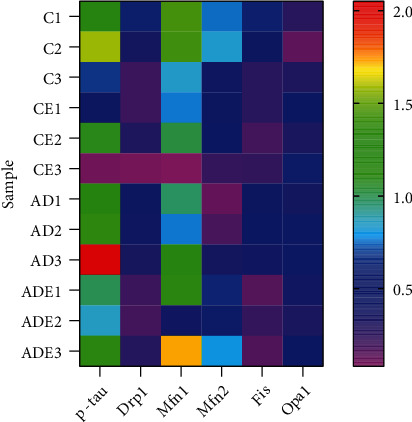
Heat map of Pearson correlation analysis between each functional parameter.

A heat map analysis of the correlation between the functional parameters is shown in Figure 7. A positive correlation was observed between the hippocampus p-TAU^ser214^ protein content and the DRP1 (*r* = 0.637, *p* < 0.05) and MFN1 (*r* = 0.617, *p* < 0.05) protein content. The DRP1 protein content was positively correlated with the p-TAU^ser214^ (*r* = 0.637, *p* < 0.05) and FIS1 (*r* = 0.841, *p* < 0.01) protein contents. The MFN2 protein content was positively correlated with the MFN1 (*r* = 0.667, *p* < 0.05) protein content and negatively correlated with the OPA1 (*r* = 0.598, *p* < 0.05) protein content.

## 4. Discussion

### 4.1. Effects of HIIT on Behavioral Testing in AD Mice

High-intensity interval training (HIIT), as a training mode with high-efficiency short bursts of exercise, has been widely been studied [14, 22, 23], yet we still have little understanding of the effects of this training intervention on neurons. The relevant research may also have had controversial results due to the diversity in the experimental designs. According to the scientific evidence, amyloid deposition and phosphorylation of tau precede cognitive impairment, brain atrophy, and loss of neuronal function in AD disease [24]. In addition, existing studies show that the neuropath logical changes in asymptomatic AD individuals are characterized by brain amyloid deposits and very subtle decline in cognitive function [25, 26]. Therefore, the measurement of cognitive performance throughout the progression of Alzheimer's disease over time should consider whether the disease is in a preclinical, prodromal, or dementia phase.

In our study, we found that AD mice displayed worse performances for cognitive function and exploratory activity relative to mice from the C group in the open-field (OF) test and elevated plus maze (EPM) tests. Specifically, AD mice had a significantly reduced locomotion area, traveled less distance, spent little time in open arms, and spent more times in the outside zone and in closed arms. However, after 10 weeks of HIIT intervention, there were significant differences in the behavioral parameters for ADE mice in both tests, such as traveled distance, maximum speed, and average speed in the OF and more time spent in the open arms in the EPM. Overall, our findings show that compared with AD mice, the ADE mice exhibited higher exploratory desire and less anxiety in an unfamiliar environment.

Considering that our findings on behavioral activity in AD mice are in line with previous studies reported in the literature, we anticipated that the brains of AD mice would show A*β* accumulation and hyperphosphorylated tau. Next, to determine whether these behavioral changes were due to the structural changes and overexpression of proteins in the hippocampus that are associated with AD or were simply due to an improvement in muscle performance resulting from HIIT, we examined the hippocampus region for morphological structural changes.

### 4.2. Effects of HIIT on Hippocampal Morphology in AD Mice

The initiation and progression of neurodegenerative diseases are complicated and related to multifaceted cellular and molecular mechanisms; therefore, it is almost impossible to define a single leading cause. At the cellular biochemical level, however, the findings of intracellular or extracellular accumulation of aberrant proteins comprising amyloid, tau, or *α*-syncline are the primary pathological hallmarks of neurodegeneration that are often associated with the abnormal handling of proteins. Generally, this injured information processing is caused by an imbalance in brain homeostasis which initially leads to a functional decline in the neural connectivity network and then subsequent structural decline with progression to trigger synaptic loss and death of nerve cells, eventually resulting in generalized brain atrophy with severe cognitive deficits. Alzheimer's disease is a neurodegenerative disease that involves multiple brain regions, such as the hippocampus (associated with memory) and the medial temporal region, as well as the cortical-associated areas in the frontal, temporal, and parietal lobes. One study on Alzheimer's patients reported that the occurrence of abnormality in the hippocampus volume precedes any other clinical symptoms measured using magnetic resonance imaging (MRI) [27], suggesting that lesions in the hippocampus play a pivotal role in the progression of Alzheimer's disease. In our research, following 10 weeks of HIIT intervention, in addition to evidence of an improvement in the arrangement and morphology of hippocampus neurons, pink amyloid plaques were significantly reduced in the hippocampuses from the ADE group. These changes indicate that long-lasting HIIT exercise for 10 weeks could contribute to alleviating the morphological and functional abnormalities in hippocampus neurons as well as in reducing the production and enhancing the clearance of amyloidosis in AD mice.

Given that glia, as an important cell component, can promote the generation, maintenance, and death of synapses and even provide metabolic support to the overall homeostasis of nervous tissue, neuron-centric theories have been challenged in the past decade, and considerable attention has been paid to neuralgia cells in the progression of brain homoeostasis. Existing evidence supports that this brain homeostasis could be fundamentally determined by atrocities which are responsible for environmental stability and defense [28]. Glial fibrillary acidic protein (GFAP), a marker of astrocytes activation, is mainly found to mediate the formation of cytoskeleton and maintenance of tension in atrocities in the central nervous system. Postmortem examination of Alzheimer's patients showed that the neuralgia were activated, with overexpression of GFAP, valentine, and S100 in the diseased brain [29]. One researcher also observed the hypertrophic reactivity of atrocities to senile plaques and per vascular A*β* deposits in AD mouse models [30]. Similarly, we clearly saw hyperplasia and hypertrophy of atrocities in both of mice from both the C group and the AD group. After 10 weeks of HIIT intervention, the above changes of atrocities were significantly improved. Hence, we inferred that systemic HIIT could restore atrocities response, including in terms of the abnormal GFAP overexpression, hyperplasia, and hypertrophy of cells, thus improving cognitive function and behavioral activity. This may be result of correcting cellular signaling events triggered by various molecules in AD disease or with aging, such as A*β*, molecules released from damaged cells, cytokines, or chemokines.

### 4.3. Effect of HIIT on Mitochondrial Kinetic and Related Proteins in Hippocampus

The brain is the most energy-intensive organ in the body, and the energy required for neuronal growth and development and message transmission is mainly produced by mitochondria. Thus, mitochondrial dysfunction may destroy high energy-demanding cells such as neurons, causing central neuropathy [31]. Besides the nucleus, mitochondria are the only other organelles in cells which contain DAN and are highly dynamic organelles that undergo coordinated cycles of fission and fusion, to maintain their distribution, shape, and size. Mitochondria play a key role in various biological processes, such as oxidative stress, energy metabolism, apoptosis, and calcium homeostasis. The biological basis for this is the dynamic changes in the balance of mitochondria, called “mitochondrial dynamics” [32, 33]. Given the important role of mitochondrial dynamics, any small alteration in fission/fusion can have catastrophic effects on mitochondrial function, energy metabolism, and redox homeostasis. Indeed, increased mitochondrial fragmentation has been observed in AD patients and animal models, suggesting an imbalance in mitochondrial dynamics [34–37]. In addition, scholars have found that the mitochondria in Alzheimer's disease patients and animal models differ from those in individuals not suffering from non-Alzheimer's disease [38].

Mitochondrial dynamic-related protein1 (DRP1) has been found to play a key role in mitochondrial division in various organisms ranging from yeast to mammals. DRP1 receptor proteins are Mff, FIS1, MiD49, and MiD51, but these receptors all have different functions. The receptor proteins MiD49 and MiD51 can function independently [35], and they appear closely related to cristae remodeling during apoptosis [36]. Regarding the role of FIS1, some scholars believe that DRP1 phosphorylation leads to an increased in FIS1 binding, causing division. However, this theory study has recently been questioned, with the suggestion that FIS1 may not interact with DRP1 but interacts with fusion proteins instead thus affecting activity [37, 39]. In this study, we found that DRP1 expression in normal aging mice and AD mice showed significantly different trends. In the normal aging mice, DRP1 expression was decreased in the CE group compared with the C group, but the difference was not significant; in the AD mice, DRP1 expression was increased in ADE group compared with AD group, but the difference was again not significant. This suggest that there may be a tendency for abnormal mitochondrial division in normal aging mice and AD mice, but that HIIT helps to ameliorate this tendency or propensity. Meanwhile, FIS1 protein expression was significantly decreased in the CE group compared with the C group. The same was seen regarding FIS1 expression in ADE mice, with the difference being highly significant compared with the AD group. Correlation analysis revealed that DRP1 and FIS1 protein contents were positively correlated. This suggests that HIIT may improve the interaction of FIS1 with the fusion protein, which in turn affects the activity of DRP1 and ameliorates the phenomenon or tendency of abnormal mitochondrial division present in AD mice and normal aging mice.

Mitochondrial fusion is also mediated by kinesis-associated GTPase proteins. Mitochondrial fusion protein 1 (MFN1) and mitochondrial fusion protein 2 (MFN2) mediate mitochondrial outer membrane fusion in vertebrates [40–42], and mitochondrial outer membrane fusion is spatially and temporally coupled to inner membrane fusion [40, 43, 44]. Endothelial fusion requires kinesis-associated basal optic nerve atrophy 1 (OPA1) [45, 46]. In this study, we found that the fusion protein expression trends in normal aging mice and AD mice were different. In normal aging mice, both mitochondrial fusion proteins MFN1 and MFN2 were decreased to different degrees in the CE group compared with the C group: MFN1 was significantly decreased, while MFN2 decreased slightly, and the difference was not significant. Regarding AD mice, the levels of both mitochondrial fusion proteins MFN1 and MFN2 were increased to different degrees in the ADE group compared with the AD group. There was a slight increase in MFN1 that was not significant, and there was a significant increase in MFN2. The protein expression of OPA1 was increased in the CE group compared with the C group, with significant differences, while OPA1 protein expression was slightly decreased in the ADE group compared with the AD group, and the difference was not significant. Correlation analysis showed that the protein content of MFN2 was positively correlated with that of MFN1 and negatively correlated with OPA1 protein content. It was revealed that the principle of abnormal mitochondrial dynamics present in normal aging mice and AD mice may differ, leading to the different effects of exercise on mitochondrial fusion proteins in mice, which suggests that the mechanisms by which HIIT improves mitochondrial fusion differ between normal aging mice and AD mice. Furthermore, morphological findings showed that 10 weeks of HIIT significantly reduced the expression of amyloid aggregates in the hippocampus in AD mice. Regarding the expression of phosphorylated tau proteins, the expression of p-TAU^ser214^ was decreased in the CE group compared with the C group, and the difference was significant, with a similar trend for the ADE group compared with the AD group. It was observed that 10 weeks of HIIT resulted in a trend of decreased phosphorylated tau protein expression, with significant difference. It is possible that, before the significant decrease in tau proteins, there were multiple manifestations of inflammation in the brain and hippocampus, causing an abnormal expression of nematic kinetic proteins. This was confirmed by the observation of GFAP expression in mouse hippocampus glial cells.

It has been reported that in AD mice or normal aging mice, inflammatory lesions may appear earlier than the pathological changes caused by the local extracellular deposition of A*β* and intracellular aggregation of tau protein/neurogenic fibrillary tangles. The exercise-induced improvement in inflammation in the relevant brain regions or tissues may equally precede the exercise-induced elimination of local extracellular deposits of A*β* and the elimination of intracellular aggregates of tau protein/neurogenic fibrillary tangles.

In the present study, HIIT exercise was found to significantly improve the tendency of mitochondrial rupture fusion in normal aging mice. However, for AD mice, 10 weeks of HIIT exercise increased the expression of both MFN1 and MFN2, with the expression of MFN2 being significantly elevated, suggesting that MFN2 is not only involved in mitochondrial fusion but may also have other roles. This is consistent with previous studies showing that MFN2 is involved in mitochondrial fusion as well as being a key factor in the mitochondrial–endoplasmic reticulum linkage site. The trend of MFN1/2 improvement in AD mice and normal aging mice as a result of 10 weeks of HIIT is different, possibly because the two are in different physiological states, resulting in different mitochondrial kinetic states or cycles, and are thus effects on different mitochondrial fusion proteins triggered by the same exercise.

## 5. Conclusion

It was found that HIIT exerted a significant neuroprotective effect, improving cognition and brain function in mice and restoring the imbalance in mitochondrial dynamics in normal aging mice and AD mice. Pathological changes precede behavioral manifestations, and therefore, the preclinical phase of AD is an important time point for exercise intervention.

## Figures and Tables

**Table 1:** Antibodies used for WB analysis of mice proteins.

Antibody name	Molecular weight (kDa)	Classification	Antibody source	Catalog number	Dilution ratio
MFN1	84	Mitochondrial fusion	BOSTER	M02172-1	1 : 1000
MFN2	86	Mitochondrial fusion	Abcam	ab124773	1 : 1000
OPA1	90–100	Mitochondrial fusion	Servicebio	GB111728	1 : 1000
DRP1	82	Mitochondrial fission	Abcam	ab184247	1 : 1000
FIS1	17	Mitochondrial fission	BOSTER	A01932-2	1 : 1000
p-TAU	50–80	Cytoplasm	Servicebio	GB13561	1 : 1000
COX IV	16	Mitochondrial	Servicebio	GB12250	1 : 1000
*β*-Actin	42	Cytoplasmic	Servicebio	GB11001	1 : 1000

## Data Availability

All data used to support the results of this study are included in the article. and the original picture used to support the findings of this study is included within the supplementary information file(s).
